# Relationships between bone age, physical performance, and motor coordination among adolescent male and female athletes

**DOI:** 10.3389/fspor.2024.1435497

**Published:** 2024-11-14

**Authors:** Hilde Gundersen, Knut Marius Navelsaker Kvammen, Mona Vestbøstad, Cecilie Brekke Rygh, Halvard Grendstad

**Affiliations:** ^1^ Department of Sport, Food and Natural Sciences, Western Norway University of Applied Sciences, Bergen, Norway; ^2^ Department of Health and Functioning, Western Norway University of Applied Sciences, Bergen, Norway; ^3^ Department of Radiology, Haukeland University Hospital, Bergen, Norway; ^4^ Department of Physical Performance, Norwegian School of Sport Sciences, Oslo, Norway

**Keywords:** puberty, skeletal age, physical capacity, motor skills, youth

## Abstract

Biological maturity significantly impacts youth athletes’ physical performance throughout adolescence. However, how this differs between male and female youth athletes remains unclear. Thus, the present study aimed to assess associations between maturity, physical performance and motor coordination in females and males. Sixty-eight youth athletes (mean age 13.9 ± 0.8 years, 26 females) were included in the present study. Participants performed a 40 m sprint, standing long jump (SLJ), push-ups and a 2,000 m run. Motor coordination was evaluated using the short form of the Körperkoordinationstest für Kinder test. Bone age (BA), assessed by x-ray of the left hand and analyzed with an automated software, was used as a biomarker of biological maturity. Results showed that BA was significantly associated with performance for males on 40 m sprint (*r* = −.556, *p* < .001), SLJ (*r* = .500, *p* < .001) and 2,000 m run (*r* = −.435, *p* = .011). No associations were found between BA and physical performance among females, nor between BA and motor coordination for either females or males. In conclusion, maturity is associated with exercises that require maximal speed, explosive leg strength and endurance in males, but not in females, with maturity showing no impact on the motor coordination in either sex.

## Introduction

During adolescence, the physical performance of male and female youth athletes may be influenced by their biological maturity (1–3). This is linked to the development of lean body mass, which influences physical performance measures, such as strength, power, and speed (4). However, biological maturity may also impact motor coordination due to changes in limb length, muscle strength, and neuromuscular control (4, 5). While the evidence supporting the effect of biological maturity on the physical performance of males is growing, the evidence for females is less conclusive and lacks scientific support (6). This uncertainty also extends to the impact of biological maturity on motor coordination in both sexes.

It is likely that the impact of maturity on physical performance and motor coordination differs between males and females, as females experience relatively smaller gains in lean body mass and a greater accumulation of fat mass compared to males (7). The few studies on females have indicated that differences in biological maturity only had minor discrepancies in the performance of sprint, agility, jump, and intermittent-endurance (6), while others have found that maturity was associated with jump performance in females (8, 9). In contrast, studies in males have shown a stronger relationship between various measures of physical performance and biological maturity (2, 3). As noted, the difference is likely due to pubertal development, with males experiencing a higher secretion of anabolic hormones (such as testosterone), resulting in larger gains in muscle mass with increasing maturity (10). Still, whether maturity has an effect on motor coordination in males is not clear. One study found that youth soccer players performed slightly better in tasks such as jumping sideways and balancing backward compared to their less mature peers (11). Nevertheless, other studies on both males and females have shown the effect of maturity to be less pronounced, with maturity only having a minimal impact on motor coordination tasks (12, 13).

The current literature has a significant limitation in that it uses various markers of biological maturity, such as anthropometric measurements and age at menarche. This could cloud our understanding of how maturity affects changes in performance during adolescence, and may explain the different results between studies. It has been suggested that bone age (BA), measured using an x-ray image of the left hand and comparing the growth plates of several bones against images from a reference atlas (14), could be the most precise measure of biological maturity (15). Unlike anthropometric estimates of maturity using height and weight, which have shown significant limitations in both males and females, BA might improve the interpretation of maturity's effect on motor coordination and physical performance for both males and females.

Therefore, to further understand the connection between maturity and performance, we conducted tests on a group of young male and female athletes to assess their bone age, physical performance, and motor coordination. We hypothesised that maturity would be more closely linked to physical performance in males than in females, and that there would be no correlation between motor coordination and maturity level in either males or females. This information could contribute to our understanding of how biological maturity should be considered when evaluating physical performance and motor coordination in youth athletes.

## Materials and methods

Data were collected in January and April 2023. All tests were conducted by experienced test personnel, and the same protocol was used on both test occasions. The study was approved by the Regional Committee for Medical and Health Research Ethics in Norway (551902) and the Norwegian Agency for Shared Services in Education and Research (SIKT) (870863) and it was conducted in accordance with the Helsinki Declaration. Since the participants were under the legal age of consent, their parents provided written informed consent for their participation.

### Participants

A total of 26 females and 42 males aged 12–15 years participated in the study. All participants were involved in sport, and 60 out of 68 indicated their sport discipline on a questionnaire. Forty-three participants engaged in a single sport, while 17 were involved in multiple sports. Most participants were engaged in team sports, with football being the most common (*n* = 35), followed by handball (*n* = 13). Among individual sports, athletics was the most frequently practiced (*n* = 8), followed by swimming and gymnastics, each with four participants. Mean weekly training sessions were 4.70 ± 0.62 (*n* = 37). For an overview of the characteristics of the participants, see Table 1.

**Table 1:** An overview of the participants characteristics.

	Females (*n* = 26)	Male (*n* = 42)
Chronological age (years)	14.0 ± 0.8 (12.6–15.1)	13.9 ± 0.8 (12.4–15.1)
Bone age (years)	13.8 ± 1.3 (11.4–16.8)	13.5 ± 1.5 (10.6–16.7)
Height (cm)	160.8 ± 6.8 (143.4–173.5)	164.1 ± 9.9 (149.3–185.4)
Weight (kg)	50.8 ± 6.9 (38.3–68.0)	51.7 ± 10.1 (34.4–85.8)

Data are represented as mean ± SD (min-max).

### Anthropometric data

Anthropometric measurements included body height and weight. Height was measured with a stadiometer (Seca 206 and Seca 217, Hamburg, Germany), and recorded to the nearest 0.1 cm. Measurements were performed barefoot using standard procedures. Weight was assessed using the InBody 720 (InBody™ 720, Biospace CO.).

### Test regime of physical performance

All participants followed the same warm-up procedure. The order of the tests was the same for all participants: 40 m sprint, standing long jump (SLJ), jumping sideways (JS), push-ups, moving sideways (MS), balancing backwards (BB) and 2,000 m running. There was approximately 10 min of rest between each test. All tests, except for the 2,000 m run, were performed indoors. Due to logistical constraints, the 2,000 m run was performed on day 2 for some participants. The participants wore a T-shirt, shorts, and sports shoes during all the tests. Due to injuries, the number of participants for each test varied.

### 40 m sprint

A 40 m linear sprint test was conducted to measure the participant's speed. After the warm-up, all participants performed three maximal sprints of 40 m, separated by 2–3 min of rest. A portable photogate system (Witty, Microgate, Italy) was used to record running time. The first photogates were positioned 50 cm above the running surface, while the photogates at 40 m were positioned 120 cm above the running surface. All participants started from a standing position with split legs, with the toes of the front foot placed 60 cm behind the first photogates. The fastest sprint of three attempts was included in the analysis.

### Standing long jump

SLJ was performed to measure explosive leg strength. Participants started with both feet placed behind a line marked on the ground and jumped as long as possible in a forward direction. The horizontal distance from the start line to the mark made by the heel was measured and used to determine the jumping length. The best of three attempts was used in the analyses.

### Push-ups

Participants started in a plank position with their hands shoulder-width apart. They then bent their elbows until touching an object on the floor with their chest. The repetition was only counted when the chest touched the object. The spine had to be in a neutral position in each repetition, and the test lasted until they failed to complete a push-up. The maximum number of approved repetitions was used in the statistical analyses.

### 2,000 m running test

Participants were instructed to run 2,000 m as fast as possible. Each participant started every 10 s, and individual running time was registered using RaceSplitter (https://www.racesplitter.com/).

### Tests of motor coordination

#### The Körperkoordinationstest für Kinder (KTK)

Motor coordination was assessed using the short form of the KTK developed by Kiphard & Schilling (16, 17). The short form KTK is comprised of three items (18): jumping sideways (JS), moving sideways (MS) and balancing backwards (BB).

### Jumping sideways

JS evaluates the bilateral symmetrical motor coordination, speed, and dynamic balance of the lower limbs. Participants jumped over a square wooden slat (60 cm × 4 cm × 2 cm) with both feet horizontally from left and right as much as possible within 15 s, two times. All participants started on a self-selected side of the wooden slat and had to jump from and land on both legs. The test score added the number of jumps between the two tries.

### Moving sideways

MS evaluates the coordination and agility of lateral movement. The test combines the velocity of the upper and lower limbs with fluidity of movement, laterality, and spatiotemporal structure. Participants stood on one of two platforms (25 cm × 25 cm × 5 cm) and moved the opposite platform by hand as fast as possible within 20 s Each participant was given two tries, one for each left and one for the right direction. The test score was the sum of the two trials.

### The balancing backwards test

BB evaluates balance control and coordination. The test was performed barefoot, and there was no time limit. Participants stepped back three times on three balance beams of different widths, each 3 m long and 5 cm high, with widths decreasing as the test progressed (6.0, 4.5, and 3.0 cm, respectively). A maximum of eight steps could be taken for each beam in each test, and a maximum of 72 steps (eight steps × three times × three beams) could be taken for the total test score. The test score was the sum of the number of test steps. The sum of backward steps was used in the analyses. The maximum score was 72.

### Skeletal maturity

Bone age (BA) was assessed using a posterior-anterior x-ray of the left hand and wrist, captured with a Siemens Ysio Max with integrated FLUORPSPOT Compacts imaging system (software version VE10; Siemens Healthineers). The x-ray field of view extended from the fingertips to 3 cm above the wrist joint, capturing the epiphyseal plates of the radius and ulna. Exposure settings were standardized at 50 kV, 1-1.5 mAs, and a 1 m tube-detector distance. No image processing or filtering was applied.

The BoneXpert standalone version 3.4.1.0 (Visiana, Holte, Denmark) was used to analyse the radiographs, based on the Greulich Pyle methodology (19). Gender as taken into consideration in the analyses. The system automatically performs 8–13 independent BA measurements from 8 to 13 different bones in the left hand. This automated process eliminates inter- and intra-observer variation. The root mean square error (RMSE) of BoneXpert is estimated to be 0.68 years in males and 0.52 years in females (20).

### Statistical analyses

Descriptive data is shown as mean ± standard deviation (SD), 95% confidence interval (CI) and minimum and maximum values. To examine the distribution of the data and assess normality, the Shapiro-Wilk test was employed. One-way ANOVA was conducted to examine differences between males and females for variables that were normally distributed and Mann Whitney-*U* test was used for not normally distributed variables. Effect sizes (*r*) were calculated to quantify the magnitude of the differences. The relationship between variables was assessed using Pearson's correlation for normally distributed data, while Spearman's rho was applied for non-normally distributed data. An *r*-value between.01 and.29 was defined as a small correlation, between 0.30 and 0.49 as a medium correlation, and from 0.5 to 1.0 as a large correlation (21). The Statistical Products of Service Solution package (SPSS, version 29) was used for all statistical analyses, and *p*-values of ≤.05 were considered as statistically significant.

## Results

### Relationships between BA, physical performance and motor coordination

Among males, there was a strong negative correlation between BA and 40 m sprint time (*r* = −.556, *p* < .001, *n* = 41), a strong positive correlation between BA and SLJ performance (*r* = .500, *p* < .001, *n* = 42), and a moderate negative correlation between BA and 2000m run time (*r* = −.435, *p* = .011, *n* = 33). Among females, there was no significant correlation between BA and physical performance, nor between BA and motor coordination. No significant correlation was found between BA and motor coordination in males either (Table 2).

**Table 2:** An overview of the relationship between bone age (BA) and physical performance in females and male.

	40 m	SLJ	Push-ups	2,000 m	JS	MS	BB
Females							
BA	*r* = −.372	*r* = .116	*r* = −.204	*r* = .168	*r* = −.064	*r* = .100	*r* = .041
	*p* = .073	*p* = .581	*p* = .351	*p* = .519	*p* = .766	*p* = .626	*p* = .843
	[−.675, .036]	[−.292, .489]	[−.577, .240]	[−.340, .600]	[−.466, .359]	[−.319, .479]	[−.363, .432]
	(*n* = 24)	(*n* = 25)	(*n* = 23)	(*n* = 17)	(*n* = 24)	(*n* = 26)	(*n* = 26)
Males							
BA	*r* = −.556	*r* = .500	*r* = .049	*r* = −.435	*r* = −.202	*r* = .144	*r* = .196
	*p* < .001	*p* < .001	*p* = .760	*p* = .011	*p* = .198	*p* = .364	*p* = .214
	[−.738, −300]	[.231, .698]	[−.262,.351]	[−.683, −.097]	[−.477, .108]	[−.177, .436]	[−.124, .479]
	(*n* = 41)	(*n* = 42)	(*n* = 41)	(*n* = 33)	(*n* = 42)	(*n* = 42)	(*n* = 42)

SLJ, standing long jump; JS, two-legged sideway jumps; MS, side-way movement; BB, balancing backward.

### Physical performance between females and males

Males did significantly more push-ups (*U* = 234.5, *p* = .001, *r* = .415) and ran faster in the 2,000 m run test (*U* = 124.5, *p* = .001, *r* = 0.452) than females. There were no other significant differences in physical performance or motor coordination abilities between males and females. For an overview of scores on the tests of physical performance and motor coordination, see Table 3. Additionally, there was no significant difference between females and males in chronological age, BA, height and weight.

**Table 3:** An overview of the performances on the physical tests.

	Females	Males
Physical capasities		
40 m sprint test (sec)	6.06 ± 0.35 (5.29–6.50) (*n* = 24)	6.00 ± 0.41 (5.03–6.65) (*n* = 41)
Standing long jump (m)	1.97 ± 0.11 (1.80–2.25) (*n* = 25)	2.05 ± 0.19 (1.76–2.54) (*n* = 42)
Push-ups (*n*)	23.4 ± 12.5 (3–63) (*n* = 23)	32.9 ± 11.9 (1–62) (*n* = 41)
2,000 m run test (min)	9.04 ± 1.03 (8.09–9.52) (*n* = 17)	8.18 ± 0.32 (6.47–11.23) (*n* = 33)
Motor coordination		
Two-legged sideway jumps (*n*)	96.2 ± 10.2 (67–114) (*n* = 24)	98.0 ± 9.0 (71–116) (*n* = 42)
Side-way movement (*n*)	31.2 ± 5.7 (12–41) (*n* = 26)	31.6 ± 6.9 (14–41) (*n* = 42)
Balancing backward (score)	67.9 ± 5.3 (50–72) (*n* = 26)	64.3 ± 9.9 (20–72) (*n* = 42)

Data are presented as mean ± SD (min-max).

## Discussion

The aim of the present study was to investigate linear relationships between biological maturity, physical performance and motor coordination in male and female adolescent athletes using seven different tests. We hypothesized that biological maturity would be more closely related to the physical performance of males than females, and that biological maturity would have less impact on motor coordination. Our results partly confirmed our hypothesis, showing that biological maturity was significantly related to maximal sprinting, jumping ability and 2,000 m endurance performance in males, but not to the number of push-ups. Furthermore, maturity was not related to any physical performance measures in females. Motor coordination performance did not show any relationship with maturity in either males or females.

The relationships between maturity and maximal sprinting and jumping performance among males align with previous findings (1–3, 22), confirming that maturity affects performance in exercises that require explosive leg power in males. Indeed, these findings are not surprising, given the hormonal changes associated with the growth spurt, which result in increased lean body mass, muscle strength, and performance in males (23). However, our research did not find any correlation between maturity and performance in push-ups among our male participants, which may be attributed to the test's procedure, as it resembles upper-body endurance performance more than maximal strength and power. This outcome aligns with a prior study on young male soccer players conducted in our lab (2).

However, the significant association we found between 2,000 m performance and maturity contrasts with other studies that have examined intermittent-endurance performance and maturity (1, 2). This relationship might be somewhat unclear. For instance, Gundersen et al. (22) found that more mature U14 soccer players (but not U15 players) performed better when results were adjusted for players height (i.e., shorter individuals with higher maturity showed better endurance performance). This may be related to the Yo-Yo IR1 intermittent-endurance performance test, which requires participants to make a 180° turn for each lap. In contrast, in our study, the 2,000 m run test was performed without turns. In general, improvements in endurance performance may be attributed to higher maximal oxygen uptake, enhanced running economy, or superior anaerobic capacity. As maximal oxygen uptake relative to body mass remains stable throughout adolescence for male youth athletes (24), it is plausible that the increased maturity observed in the males in our study has contributed to better running economy and/or anaerobic capacity, thereby translating to improved performance in the 2,000 m event (1, 2, 25). We did not find any significant relationship between maturity and physical performance in females. Only a few previous studies have investigated the relationship between maturity and physical performance in females. Two studies have found that both vertical jump performance (4, 24) and standing long jump (26) were significantly related to maturity in youth female athletes, based on equations from anthropometric variables to estimate peak height velocity (PHV). Similar findings were also observed for 30 m sprint performance (27). Based on these studies, more mature females performed better in jumping and sprinting tasks, which contradicts our findings. However, the physical performance differences between females and males may be attributed to the methods used to estimate maturity. Estimates of PHV for females are less accurate, and utilizing BA improves our interpretation of the relationship between maturity and physical performance. While it is likely that neuromuscular function changes during growth and maturation contribute to improved jumping and sprinting performance (28), the gains in fat mass for females may obscure physiological changes during weight-bearing activities like running and jumping. Previous studies using longitudinal data suggest that jump and sprint performance in females peaks around 13–14 years of age before leveling off (29–31). This is also evident in the development of maximal oxygen uptake (relative to body mass), which has been shown to decrease as females mature throughout adolescence (25). More research utilizing BA as a maturity marker is necessary to better understand how maturity impacts females’ physical performance.

Our study found no correlation between biological maturity and motor coordination in either males or females, indicating that maturity is not linked to motor coordination. Our results align with a recent study that explored the relationship between bone age and motor performance in females. That study found that bone age accounted for only 1.8% and 5.8% of the variance in motor performance among females aged 10–12 and 13–15, respectively (32). Meanwhile, a previous study of male soccer players aged 5–19 showed that the development of motor competence occurred before the adolescent growth spurt in males (33). This suggests that motor coordination may develop at an earlier age in males than in our study population. As the JS test involves hopping and jumping, which may also require skills such as strength and speed to perform well, we might have expected a relationship between maturity level and the two-legged sideway jump test. On the other hand, the jumping sideway test lasts 2 × 15 s and is not a measure of maximal explosive strength, which previously was associated with maturity level in males. Furthermore, it is possible that the age of the participants and the athletic experience of our research subjects limited the potential influence of BA on motor coordination assessment. This is supported by findings from a study conducted by O’Brien Smith and colleagues in 2019 (34), which revealed that motor coordination abilities may be a persistent trait among highly skilled adolescent athletes. To explore this further, future studies should also include younger athletes in their cohort.

This study aimed to comprehensively assess physical performance and motor coordination in both male and female subjects. However, certain limitations must be considered. Firstly, the age of our sample may have been too high to reveal strong relationships between biological age and physical performance and/or motor coordination, especially in females who reach puberty earlier than males. In line with this, the females in our study likely had a bone age reflecting that they had already gone through menarche. As this is a late maturational event, it may have affected the relationship between physical performance and biological maturation in the females. Additionally, the sample size on some tests was small, particularly for females, which could have limited the statistical power. Future studies should aim to include younger age groups to represent the entire maturity continuum.

Our findings confirmed the hypothesis that biological maturity has a greater influence on physical performance in young males than in young females. However, no significant correlation was found between motor coordination and maturity level in either sex.

### Practical implications

This study highlights the significance of accounting for biological maturity in evaluation of physical performance, particularly for activities that require strength and explosive effort in adolescent males. Conversely, our results suggest that the biological maturity in females requires a different level of consideration. However, biological maturity does not affect motor coordination tasks in either sex, implying that these assessments can be conducted without the influence of biological maturity. The results of this study and previous research suggest that motor coordination tests could prove useful in identifying the motor skills of young adolescent athletes, irrespective of biological maturity. Thus, these assessments may have practical implications for coaches and practitioners seeking to identify promising young athletes and facilitate their development.

## Data Availability

The raw data supporting the conclusions of this article will be made available by the authors, without undue reservation.
